# EFFECTS OF IZUMIOTSU CITY’S COGNITIVE DECLINE PREVENTION DANCE AS A STRUCTURED ACTIVITY IN ADULT DAY SERVICES

**DOI:** 10.1093/geroni/igae098.3962

**Published:** 2024-12-31

**Authors:** Atsuko Miyazaki, Kaori Ueki, Atsushi Hiyama

**Affiliations:** The University of Tokyo, Tokyo, Japan; TOPPAN Inc., Tokyo, Japan; Hitotsubashi University, Kunitachi, Tokyo, Japan

## Abstract

This study examined the effects of an innovative cognitive decline prevention dance program in adult day services. Developed through a community co-creation living lab in Izumiotsu City, Japan, this Hip-Hop-inspired dance is specifically designed for individuals with mild cognitive impairment (MCI), who often struggle with imitation tasks. The program replaced 40 minutes of a 2-hour high-intensity circuit training session in day services. Twenty-six day service users (3 males, 23 females; mean age 82.62 years, SD=4.09) participated in a 4-month intervention study, divided into a dance group (n=14) and a fitness group (n=12, continuing full circuit training). Results showed significant improvements in the dance group compared to the fitness group in cognitive function tasks, including arithmetic operations (F(1,21) = 5.65, p = 0.018, η2 = 0.205) and spatial memory accuracy (F(1,21) = 4.23, p = 0.036, η2 = 0.168). Postural assessments revealed significant improvements in the dance group, including increased shoulder height symmetry (F(1,21) = 3.53, p = 0.046, η2 = 0.143) and reduced left knee hyperflexion in standing position (F(1,21) = 19.19, p = 0.001, η2 = 0.477), indicating better postural alignment. This dance program is a product of industry-academia-government-civil collaboration, combining university research, municipal policy-making, corporate development, and citizen participation. As a result of this collaborative approach, an effective program tailored to the needs of older adults was created. The significant improvements observed suggest that innovating structured activities in adult day services, through such collaborative efforts, offers a promising approach to addressing health challenges in super-aged societies.

